# The value of serum Mac-2 binding protein glycosylation isomer in the diagnosis of liver fibrosis: a systematic review and meta-analysis

**DOI:** 10.3389/fphys.2024.1382293

**Published:** 2024-10-30

**Authors:** Xinyu Liu, Wei Zhang, Baofeng Ma, Chunlei Lv, Mimi Sun, Qinghua Shang

**Affiliations:** ^1^ College of First Clinical Medicine, Shandong University of Traditional Chinese Medicine, Jinan, Shandong, China; ^2^ Department of Liver Disease, The 960th Hospital of the PLA Joint Logistics Support Force, Jinan, China; ^3^ The third department of encephalopathy, Jinan Integrated Traditional Chinese and Western Medicine Hospital, Jinan, China; ^4^ Diagnosis and Treatment Center for Liver Diseases, Tai’an 88 Hospital, Taian, China

**Keywords:** mac-2-binding protein glycosylation isomer, liver fibrosis, diagnosis, meta-analysis, non-invasive diagnostic index

## Abstract

**Background:**

The early detection and intervention of liver fibrosis (LF) in patients with chronic liver disease is critical to their management. The accuracy of serum Mac-2 binding protein glycosylation isomer (M2BPGi) in the diagnosis of LF remains controversial. This study aimed to comprehensively assess the value of serum M2BPGi in diagnosing LF.

**Methods:**

The PubMed, Embase, MEDLINE, Web of Science, and Cochrane Library databases were searched. The effect values were combined using a random-effects model. Meta-regression and subgroup analysis were used to explore the sources of heterogeneity. In addition, publication bias assessment and sensitivity analysis were conducted.

**Results:**

This study includes 12 studies with 2,416 patients. The pooled sensitivity, specificity, and AUROC of M2BPGi in the diagnosis of significant fibrosis (≥F2) were 0.65 (95% CI: 0.57–0.71), 0.79 (95% CI: 0.72–0.84), and 0.78 (95% CI: 0.74–0.81), respectively, while those for predicting extensive fibrosis (≥F3) were 0.76 (95% CI: 0.71–0.80), 0.75 (95% CI: 0.68–0.81), and 0.81 (95% CI: 0.77–0.84). Sensitivity analysis indicated stable results in this study. The disease type, cut-off values, study country, average age, and male proportion were the sources of heterogeneity in diagnosing significant fibrosis of M2BPGi (*p* < 0.05). Sample size, disease type, study country, publication year, cut-off values, average age, and male proportion were important sources of heterogeneity in diagnosing extensive fibrosis (*p* < 0.05).

**Conclusion:**

Serum M2BPGi has good diagnostic performance for significant fibrosis and extensive fibrosis in patients with chronic hepatitis B (CHB), chronic hepatitis C (CHC), or nonalcoholic fatty liver disease (NAFLD) and is an effective, non-invasive, and convenient marker.

**Systematic Review Registration:**

https://inplasy.com/inplasy-2023-10-0086/.

## Introduction

Liver fibrosis (LF) is a necessary stage in the progression of chronic liver disease (CLD) of various causes. If not intervened in time, it can further develop into cirrhosis, liver cancer, and eventually liver failure (Chen et al., 2022; Khanam et al., 2021). Accurate assessment of the extent of LF in patients with CLD is critical to their management. Hepatic histological examination is considered the gold standard for the diagnosis of LF, but it is an invasive operation with potential complications and low acceptance. Therefore, it is difficult to apply for dynamic monitoring of the progression of LF. Therefore, it is necessary to find effective and convenient non-invasive tests for staging and monitoring LF.

Although biomarkers such as Golgi protein 73 (GP73), ceruloplasmin, and chitinase 3-like protein 1 (CHI3L1, also known as YKL-40) have been proposed, their diagnostic accuracy remains to be confirmed (Liu et al., 2018; Kang et al., 2020; Yan et al., 2018). Mac-2 binding protein (M2BP), a secreted glycoprotein present in the extracellular matrix, is associated with cell adhesion (Lian et al., 2022). Mac-2 binding protein glycosylation isomer (M2BPGi), also known as serum wisteria floribunda agglutinin (WFA) positive Mac-2 binding protein (WFA^+^-M2BP), is a novel marker for assessing LF, which was first proposed by Kuno et al. (2013). In their study, six candidate lectins were selected for binding to M2BP, with WFA being superior to the other five for diagnosing LF (Ishii et al., 2017). Bekki et al. (2017) found that hepatic stellate cells (HSCs) are the source of M2BPGi in subpopulations of liver-derived cells. Another study showed that M2BPGi is a messenger sent by HSCs to Kupffer cells during LF (Shirabe et al., 2018). These findings indicated that M2BPGi plays an important role in the progression of fibrosis.

M2BPGi has been increasingly used in clinical applications for diagnosing LF in chronic hepatitis B(CHB), chronic hepatitis C (CHC), nonalcoholic fatty liver disease (NAFLD), and for predicting hepatocellular carcinoma (HCC) risk in these diseases (Tamaki et al., 2021; Morio et al., 2017; Fujita et al., 2018; Inoue et al., 2018; Nishikawa et al., 2016a; Atsukawa et al., 2018). Furthermore, it has been reported that M2BPGi can also be used for the diagnosis of LF in autoimmune liver disease (Nishikawa et al., 2016b; Migita et al., 2018; Umemura et al., 2015; Nishikawa et al., 2016c; Umetsu et al., 2018) and biliary atresia (Ueno et al., 2018). However, systematic reviews regarding the accuracy of M2BPGi in staging LF are relatively rare and need to be further enriched. Therefore, we provide an updated meta-analysis, not limited to LF patients with one single cause, to better assess the diagnostic performance of serum M2BPGi in staging LF.

## Methods

### Literature search

This study followed the Preferred Reporting Items for Systematic Reviews and Meta-Analysis (PRISMA) guidelines with the registration number INPLASY2023100086 (Liberati et al., 2009). The PubMed, Embase, MEDLINE, Web of Science, and Cochrane Library databases were searched. The search period was from the start of database construction until 8 September 2023. Search terms were set as follows: (‘Mac-2 binding protein glycosylation isomer’ OR (‘Mac 2’ AND (‘binding’/exp OR binding) AND (‘protein’/exp OR protein) AND (‘glycosylation’/exp OR glycosylation) AND (‘isomer’/exp OR isomer)) OR ‘wisteria floribunda agglutinin-positive Mac-2-binding protein’ OR ((‘wisteria’/exp OR wisteria) AND (‘floribunda’) AND (‘agglutinin positive’) AND (‘Mac 2 binding’) AND (‘protein’/exp OR protein)) OR ‘wfa M2bp’ OR, (‘wfa’, AND ‘M2bp’), OR ‘M2bpgi’, OR ‘wfa-binding M2bp’ OR (‘wfa binding’ AND ‘M2bp’), OR ‘wfa + M2bp’)) AND (‘liver diseases’ OR ‘liver’ OR ‘hepatic’) NOT ‘animals’ NOT (‘humans’ AND ‘animals’).

### Inclusion and exclusion criteria of the literature

Literature inclusion criteria: (1) Patients aged ≥18 years; (2) A precise classification of LF using pathological diagnosis as the gold standard; (3) Patients tested for M2BPGi; and (4) The number of direct or indirect true positives (TP), false positives (FP), true negatives (TN), or false negatives (FN) identified by M2BPGi in each case group.

Exclusion criteria for literature: (1) Animal experiments, cell experiments, case reports, conference abstracts, reviews, comments, or letters; (2) Patients without a clear diagnosis based on liver biopsy; (3) Sample size less than ten cases; (4) Inability to extract key data; (5) Duplication of published data; and (6) Full text of literature not found.

### Data extraction

The study data were extracted by two researchers independently using the pre-defined data extraction form, and any disagreements were resolved by the third reviewer. The following data were extracted: first author, publication year, disease type, fibrosis stage, study location, study design, gold standard methodology, staging criteria of LF, cut-off value, sample size, sensitivity, specificity, TP, FP, FN, and TN.

### Evaluation of quality

Two researchers independently assessed the quality of the included studies using the Quality Assessment of Diagnostic Accuracy Studies (QUADAS-2) tool (Whiting et al., 2011). Disagreements were resolved through consultation. The results of the quality assessment were presented using Review Manager software (Version 5.3.5; Nordic Cochrane Centre; Copenhagen, Denmark).

### Definition of LF

LF was staged according to the Nonalcoholic Steatohepatitis (NSH) Clinical Research Network (CRN) system, Brunt’s criteria, and the Metavir scoring system (Goodman, 2007; Bedossa and Poynard, 1996; Kleiner et al., 2005). The scoring methods of the included studies were translated into uniform criteria for staging LF. Significant fibrosis and extensive fibrosis were defined as stages ≥ F2 and ≥ F3, respectively.

### Statistical analysis

All data analysis and image generation were carried out using Stata software (version 14.0; Stata Corp LP; Texas, United States) and Meta-Disc 1.4. The pooled values of sensitivity, specificity, positive likelihood ratio (PLR), negative likelihood ratio (NLR), diagnostic score, diagnostic odds ratio (DOR), and the summary area under the curve (sAUC) were summarized using the “MIDAS” module. Diagnostic accuracy was evaluated using the sAUC. The Meta-Disc software was used to test the threshold effect, which was assessed by the Spearman correlation coefficient. Heterogeneity between studies was assessed using Cochran’s Q statistic) in Stata. A fixed-effects method was used when I^2^ < 50 or the *p*-value was <0.10. If not, a random-effects model was suitable. Meta-regression and subgroup analysis were used to explore the sources of heterogeneity. Sensitivity analysis was used to assess the influence of individual studies on heterogeneity and to observe the stability of the summary statistics. Publication bias was assessed using Deeks’ funnel plot. A two-tailed *p* < 0.05 was regarded as statistically significant.

## Results

### Search results

**FIGURE 1:**
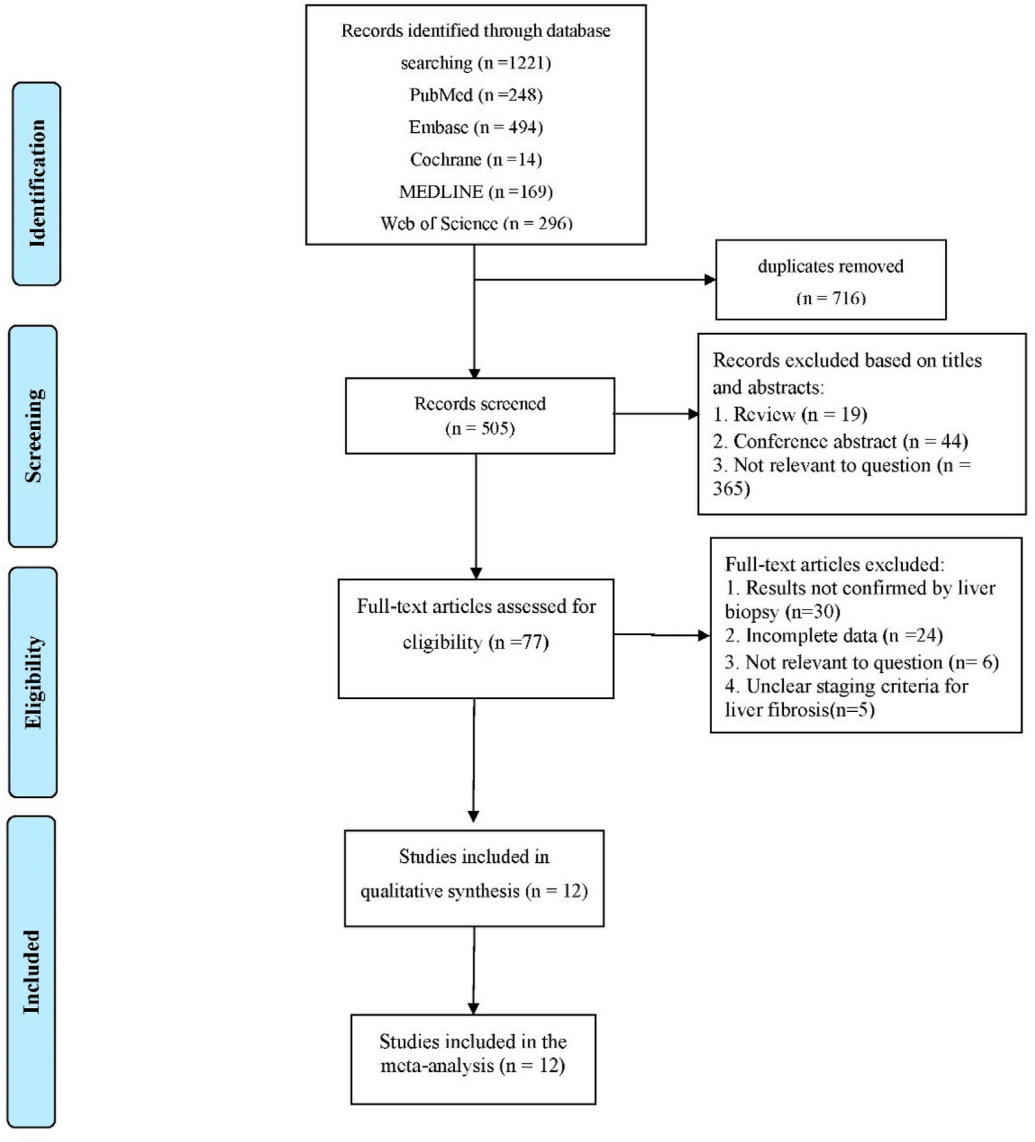
PRISMA flowchart of the study selection.

A total of 1,221 articles were retrieved after the selection process (Figure 1). After successively checking for duplicates, reading titles and abstracts, and reading the full text, 12 articles were finally included in the analysis (Nishikawa et al., 2016a; Atsukawa et al., 2018; Yeh et al., 2019; Nishikawa et al., 2016d; Abe et al., 2015; Lai et al., 2017; Huang et al., 2017; Jang et al., 2021; Tsuji et al., 2020; Numao et al., 2021; Noguchi et al., 2017; Jang et al., 2022).

### Study characteristics and quality

**FIGURE 2:**
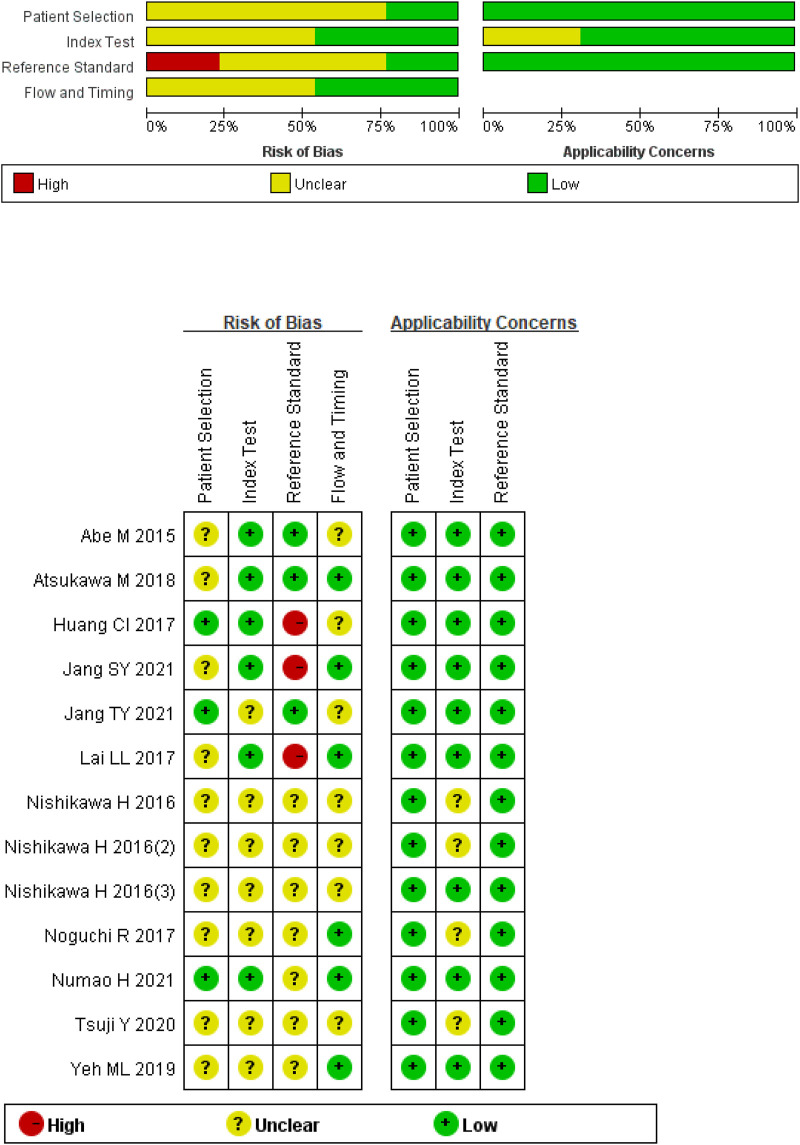
Quality assessment of included studies based on the Quality Assessment of Diagnostic Accuracy Studies tool criteria.

This study includes 12 studies with a total of 2,416 patients with CHB, CHC, or NAFLD. The gold standard for staging LF in all studies was liver biopsy. The cut-off value of M2BPGi for the diagnosis of each LF stage was set in various studies. Basic patient characteristics are summarized in Table 1. The results of the quality assessment based on the QUADAS-2 tool are shown in Figure 2.

**TABLE 1:** Characteristics of the included studies.

First author	Disease type	Fibrosis stage	Study location	Gold standard	Criteria	Sample size (n)	Study design	Cut-off	AUC	SEN (%)	SPE (%)	TP	FP	FN	TN
Yeh et al. (2019)	CHB	F2–4	Taiwan	Liver biopsy	Metavir	160	Retrospective	1.345	0.78	65.9	80.6	58	14	30	58
	CHB	F3–4	Taiwan	Liver biopsy	Metavir	160	Retrospective	1.535	0.785	66.7	79.8	34	22	17	87
Nishikawa et al. (2016d)	CHB	F2–4	Japan	Liver biopsy	Metavir	249	Retrospective	1.37	0.728	60.4	74.6	67	35	44	103
	CHB	F3–4	Japan	Liver biopsy	Metavir	249	Retrospective	1.42	0.721	66.7	69.3	40	58	20	131
	CHC	F2–4	Japan	Liver biopsy	Metavir	386	Retrospective	2.42	0.801	62.2	88.3	171	13	104	98
	CHC	F3–4	Japan	Liver biopsy	Metavir	386	Retrospective	2.03	0.83	75.5	77.6	160	39	52	135
Abe et al. (2015)	NAFLD	F2–4	Japan	Liver biopsy	Brunt’s criteria	325	Retrospective	0.9	0.838	77.3	88.1	109	18	32	130
	NAFLD	F3–4	Japan	Liver biopsy	Brunt’s criteria	325	Retrospective	0.94	0.876	85.9	74.6	79	50	13	147
Lai et al. (2017)	NAFLD	F2–4	Malaysia	Liver biopsy	NASH-CRN	220	Prospective	0.66	0.71	59.3	75.2	95	15	66	44
	NAFLD	F3–4	Malaysia	Liver biopsy	NASH-CRN	220	Prospective	0.69	0.74	62.8	75.7	27	43	16	134
Nishikawa et al. (2016a)	NAFLD	F2–4	Japan	Liver biopsy	Brunt’s criteria	134	Retrospective	1	0.663	47.2	78.6	50	6	56	22
	NAFLD	F3–4	Japan	Liver biopsy	Brunt’s criteria	134	Retrospective	1.1	0.842	73.7	80.2	28	19	10	77
Atsukawa et al. (2018)	NAFLD	F2–4	JAPAN	Liver biopsy	Brunt’s criteria	213	Retrospective	0.94	0.784	77.9	75	60	34	17	102
	NAFLD	F3–4	JAPAN	Liver biopsy	Brunt’s criteria	213	Retrospective	1.23	0.825	75	79.2	30	36	10	137
Huang et al. (2017)	CHC	F2–4	Taiwan	Liver biopsy	Metavir	229	Prospective	1.61	0.624	67.4	52.9	97	40	47	45
	CHC	F3–4	Taiwan	Liver biopsy	Metavir	229	Prospective	1.42	0.584	71.6	41.8	63	82	25	59
Jang et al. (2021)	NAFLD	F2–4	Korea	Liver biopsy	NASH-CRN	113	Retrospective	0.65	0.819	70.5	84.6	42	8	18	45
	NAFLD	F3–4	Korea	Liver biopsy	NASH-CRN	113	Retrospective	0.71	0.866	85.3	78.5	28	17	5	63
Tsuji et al. (2020)	CHB	F2–4	JAPAN	Liver biopsy	Metavir	96	Retrospective	0.89	0.902	92.6	82.4	25	12	2	57
	CHB	F3–4	JAPAN	Liver biopsy	Metavir	96	Retrospective	0.77	0.865	100	63.4	13	30	0	53
Numao et al. (2021)	CHC	F2–4	JAPAN	Liver biopsy	Metavir	141	Retrospective	2.2	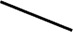	65.8	82.3	51	11	26	53
	CHC	F3–4	JAPAN	Liver biopsy	Metavir	141	Retrospective	2.99	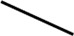	76.1	93.3	38	6	12	85
Noguchi et al. (2017)	CHB	F2–4	Japan	Liver biopsy	Metavir	70	Retrospective	0.81	0.717	50	48.9	18	17	18	17
	CHB	F3–4	Japan	Liver biopsy	Metavir	70	Retrospective	0.82	0.791	87.5	64	17	18	2	33
Jang et al. (2022)	NAFLD	F2–4	Taiwan	Liver biopsy	Metavir	80	Prospective	1.63	0.56	31	88.2	9	6	20	45
	NAFLD	F3–4	Taiwan	Liver biopsy	Metavir	80	Prospective	1.37	0.83	75	79.4	9	14	3	54

NASH-CRN, non-alcoholic steatohepatitis (NASH), Clinical Research Network (CRN); SEN, sensitivity; SPE, specificity; TP, true positive; FP, false positive; FN, false negative; TN, true negative.

### Heterogeneity

**FIGURE 3:**
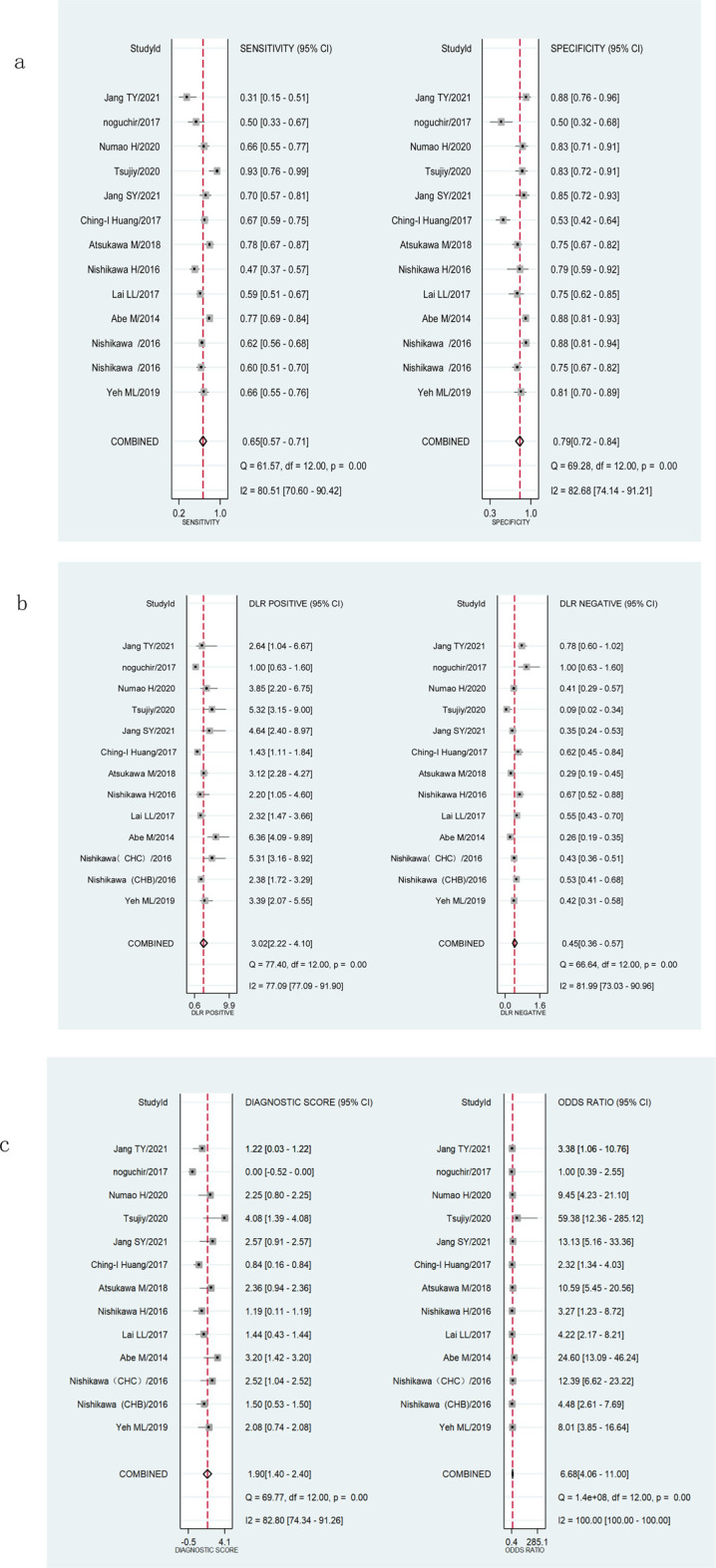
Forest plots showing the pooled evaluation indicators of M2BPGi for staging significant fibrosis (≥F2). **(A)** Pooled sensitivity and specificity. **(B)** Pooled positive likelihood ratio and negative likelihood ratio. **(C)** Pooled diagnostic score and diagnostic odds ratio.

**FIGURE 4:**
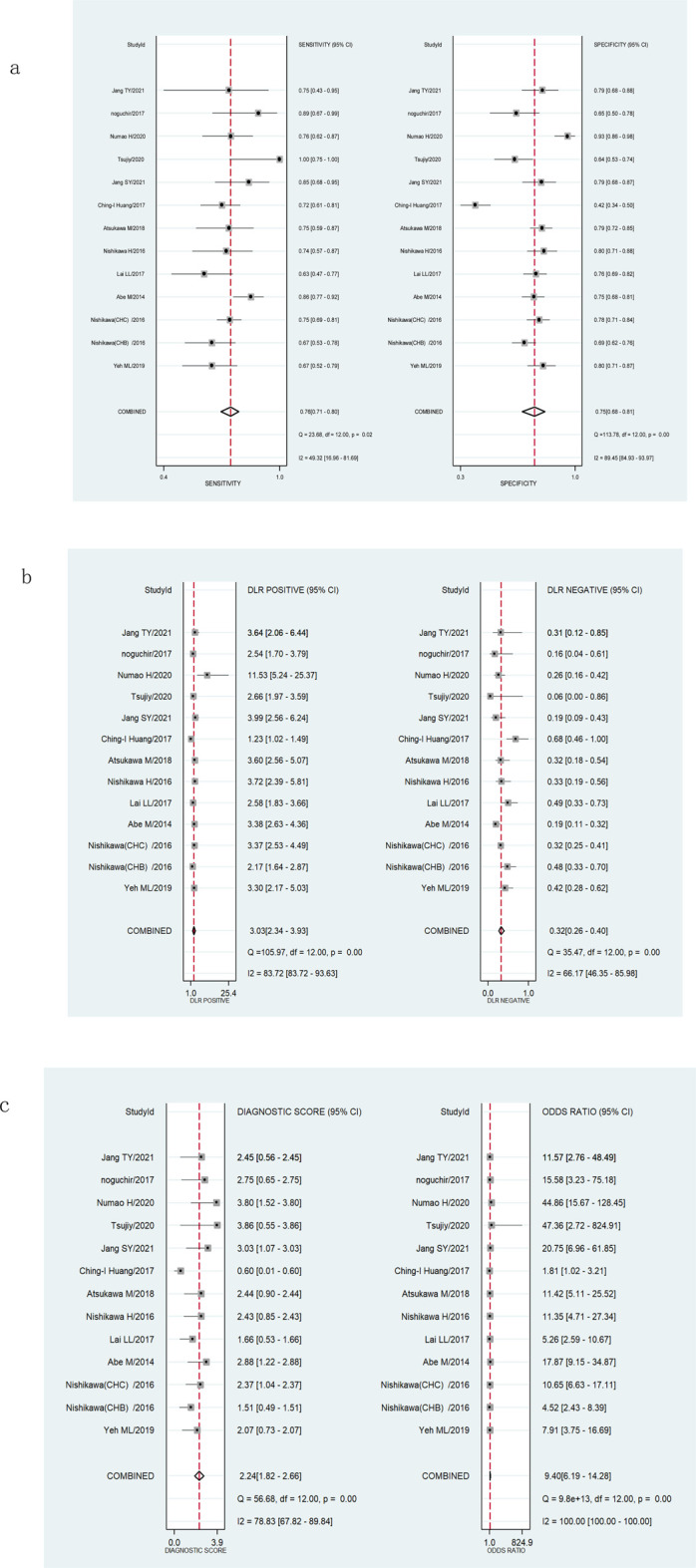
Forest plots showing the pooled evaluation indicators of M2BPGi for staging extensive fibrosis (≥F3). **(A)** Pooled sensitivity and specificity. **(B)** Pooled positive likelihood ratio and negative likelihood ratio. **(C)** Pooled diagnostic score and diagnostic odds ratio.

The Spearman’s correlation analysis showed that the correlation coefficient of M2BPGi was −0.132 (*p* = 0.668) for staging significant fibrosis (≥F2) and 0.206 (*p* = 0.499) for staging extensive fibrosis (≥F3), indicating no heterogeneity due to threshold effects. The I2 for sensitivity, specificity, PLR, NLR, diagnostic score, and DOR were all >50% (*p *<0.001) (Figures 3A–C; Figures 4A–C), suggesting the existence of a non-threshold impact due to inter-literature heterogeneity. Therefore, the effect values were combined using the random-effects model.

### Diagnostic accuracy of M2BPGi for staging LF

**FIGURE 5:**
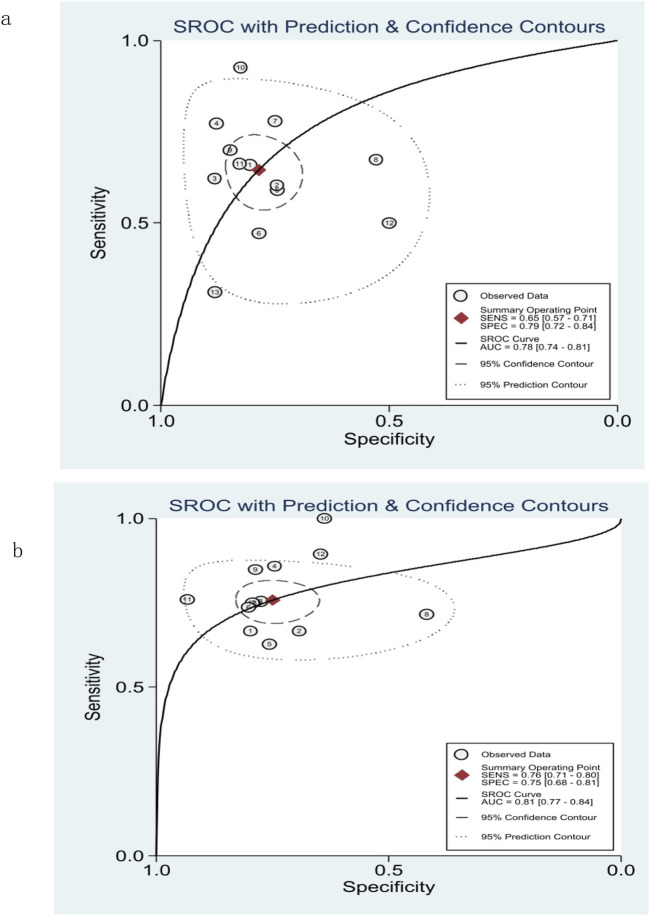
Summary receiver operating characteristic (SROC) curve of M2BPGi for predicting significant fibrosis (≥F2) **(A)** and extensive fibrosis (≥F3) **(B)**.

The pooled sensitivity, specificity, and AUROC of M2BPGi in the diagnosis of significant fibrosis (≥F2) were 0.65 (95% CI: 0.57–0.71), 0.79 (95% CI: 0.72–0.84), and 0.78 (95% CI: 0.74–0.81), respectively, (Figure 3A; Figure 5A), while those for predicting extensive fibrosis (≥F3) were 0.76 (95% CI: 0.71–0.80), 0.75 (95% CI: 0.68–0.81), and 0.81 (95% CI: 0.77–0.84) (Figure 4B; Figure 5B). The PLR, NLR, diagnostic score, and DOR for diagnosing significant fibrosis (≥F2) were 3.02 (95% CI: 2.22–4.10), 0.45(95% CI: 0.36–0.57), 1.90(95% CI: 1.40–2.40), and 6.68 (95% CI: 4.06–11.00), respectively (Figures 3B, C). The values for staging extensive fibrosis (≥F3) were 3.03 (95% CI: 2.34–3.93), 0.32 (95% CI: 0.26–0.40), 2.24 (95% CI: 1.82–2.66), and 9.40 (95% CI: 6.19–14.28), respectively (Figures 4B, C).

### Subgroup analysis and meta-regression

**FIGURE 6:**
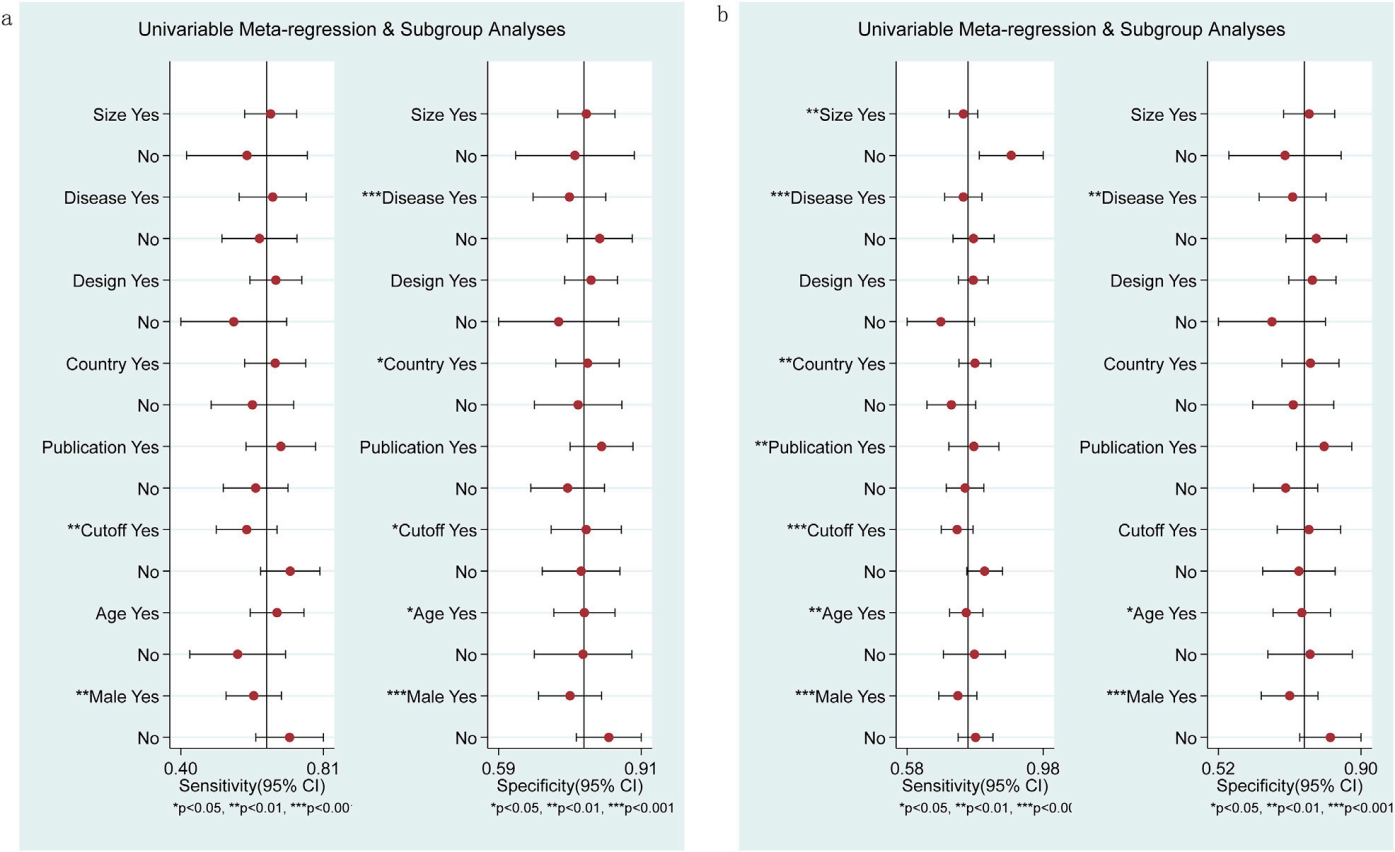
Subgroup and meta-regression analyses for M2BPGi in the diagnosis of significant fibrosis (≥F2) **(A)** and extensive fibrosis (≥F3) **(B)**.

Sources of heterogeneity were analyzed using meta-regression and subgroup analysis. Eight covariables were included: sample size (≥100 cases or <100 cases), disease type (chronic viral hepatitis or NAFLD), study design (prospective or retrospective studies), study location (Japan or not Japan), publication year (2014–2017 or 2018–2023), cut-off value (≥1 or <1), average age (≥50 or <50), and male proportion (≥0.5 or <0.5). The results indicated that male proportion and cut-off value were the sources of sensitivity heterogeneity in diagnosing significant fibrosis (≥F2) in M2BPGi (P<0.01), while disease type, study location, cut-off value, average age, and male proportion were important sources of specificity heterogeneity (Figure 6A). Sample size, disease type, study country, publication year, cut-off value, average age, and male proportion were important sources of sensitivity heterogeneity in diagnosing extensive fibrosis (≥F3), while disease type, average, and male proportion were important sources of specificity heterogeneity (Figure 6B).

### Publication bias

**FIGURE 7:**
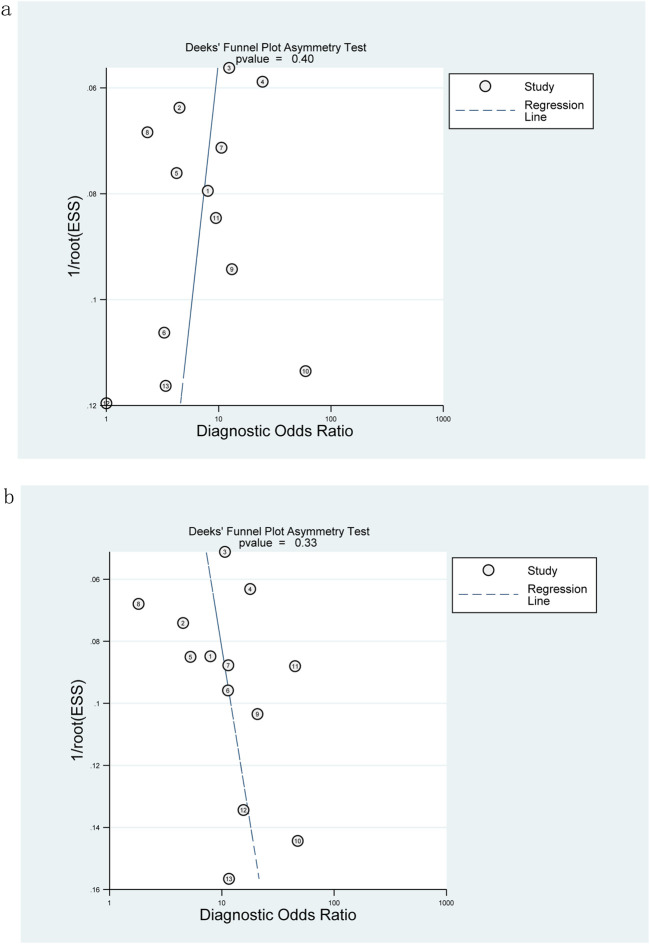
Deeks funnel plot for evaluating publication bias: M2BPGi was used to detect significant fibrosis (≥F2) **(A)** and extensive fibrosis (≥F3) **(B)**.

The Deeks funnel plot showed no significant publication bias in the included studies diagnosing significant fibrosis (≥F2) (*p* = 0.4, Figure 7A) and extensive fibrosis (≥F3) (*p* = 0.33, Figure 7B).

### Sensitivity analysis

The sensitivity analyses, performed by eliminating each study individually, showed no significant changes in overall diagnostic values, sensitivity, specificity, and their 95% confidence intervals (95% CIs), which were within the 95% CIs of the total combination values, indicating stable results in this study (Tables 2, 3).

**TABLE 2:** Sensitivity analysis for M2BPGi in the diagnosis of significant fibrosis (≥F2)**.**

Studies omitted (≥F2)	SEN (95% CI)	SPE (95% CI)	sAUC (95% CI)
Yeh et al. (2019)	0.64 (0.56–0.72)	0.78 (0.71–0.84)	0.78 (0.74–0.81)
Nishikawa et al. (2016d)	0.65 (0.57–0.72)	0.79 (0.72–0.85)	0.79 (0.75–0.82)
Nishikawa et al. (2016d)	0.65 (0.56–0.72)	0.77 (0.71–0.83)	0.78 (0.74–0.81)
Abe et al. (2015)	0.63 (0.55–0.70)	0.78 (0.71–0.83)	0.77 (0.73–0.80)
Lai et al. (2017)	0.65 (0.57–0.73)	0.79 (0.72–0.85)	0.79 (0.75–0.82)
Nishikawa et al. (2016a)	0.66 (0.59–0.73)	0.79 (0.72–0.84)	0.78 (0.72–0.82)
Atsukawa et al. (2018)	0.63 (0.56–0.70)	0.79 (0.72–0.85)	0.79 (0.73–0.81)
Huang et al. (2017)	0.64 (0.56–0.72)	0.80 (0.75–0.85)	0.80 (0.77–0.84)
Jang et al. (2021)	0.64 (0.56–0.72)	0.78 (0.71–0.84)	0.78 (0.74–0.81)
Tsuji et al. (2020)	0.63 (0.56–0.69)	0.78 (0.71–0.84)	0.75 (0.71–0.79)
Numao et al. (2021)	0.64 (0.56–0.72)	0.78 (0.71–0.84)	0.78 (0.74–0.81)
Noguchi et al. (2017)	0.65 (0.58–0.73)	0.80 (0.75–0.85)	0.80 (0.77–0.84)
Jang et al. (2022)	0.66 (0.60–0.73)	0.78 (0.71–0.83)	0.77 (0.73–0.81)
Overall	0.65 (0.57–0.71)	0.79 (0.72–0.84)	0.78 (0.74–0.81)

CI, confidence interval; SEN, sensitivity; SPE, specificity; sAUC, summary area under the curve.

**TABLE 3:** Sensitivity analysis for M2BPGi in the diagnosis of extensive fibrosis (≥F3)**.**

Studies omitted (≥F2)	SEN (95% CI)	SPE (95% CI)	sAUC (95% CI)
Yeh et al. (2019)	0.77 (0.72–0.81)	0.75 (0.67–0.81)	0.81 (0.78–0.85)
Nishikawa et al. (2016d)	0.77 (0.72–0.81)	0.76 (0.68–0.82)	0.82 (0.78–0.85)
Nishikawa et al. (2016d)	0.76 (0.70–0.81)	0.75 (0.67–0.81)	0.82 (0.78–0.85)
Abe et al. (2015)	0.74 (0.70–0.77)	0.75 (0.68–0.81)	0.75 (0.71–0.79)
Lai et al. (2017)	0.77 (0.72–0.81)	0.75 (0.68–0.81)	0.81 (0.78–0.84)
Nishikawa et al. (2016a)	0.76 (0.71–0.81)	0.75 (0.67–0.81)	0.81 (0.78–0.85)
Atsukawa et al. (2018)	0.76 (0.71–0.81)	0.75 (0.67–0.81)	0.81 (0.78–0.84)
Huang et al. (2017)	0.77 (0.71–0.82)	0.77 (0.72–0.81)	0.84 (0.80–0.87)
Jang et al. (2021)	0.76 (0.71–0.80)	0.75 (0.68–0.81)	0.81 (0.77–0.84)
Tsuji et al. (2020)	0.75 (0.71–0.79)	0.76 (0.69–0.82)	0.80 (0.77–0.84)
Numao et al. (2021)	0.76 (0.71–0.81)	0.73 (0.67–0.78)	0.81 (0.77–0.84)
Noguchi et al. (2017)	0.75 (0.71–0.79)	0.76 (0.69–0.82)	0.81 (0.77–0.84)
Jang et al. (2022)	0.76 (0.71–0.80)	0.75 (0.67–0.81)	0.81 (0.77–0.84)
Overall	0.76 (0.71–0.80)	0.75 (0.68–0.81)	0.81 (0.77–0.84)

CI, confidence interval; SEN, sensitivity; SPE, specificity; sAUC, summary area under the curve.

## Discussion

Early detection and intervention of LF in patients with CLD can reduce the risk of fibrosis-related complications and HCC. The promotion of non-invasive, simple, and effective tests can be used with more LF patients who refuse liver biopsy. In this meta-analysis, we included 12 studies on LF with causes of CHB, CHC, or NAFLD and synthesized the accuracy of M2BPGi in staging LF. Although it has been reported that M2BPGi can also be used for the diagnosis of LF with other causes (such as autoimmune hepatitis (Nishikawa et al., 2016b; Migita et al., 2018) or primary biliary cholangitis (Umemura et al., 2015; Nishikawa et al., 2016c)), these studies were excluded for small sample size or absence of liver biopsy, the gold standard for staging LF. In this study, the AUROC values for significant fibrosis (≥F2) and extensive fibrosis (≥F3) were 0.78 and 0.81, respectively. In general, an AUROC of 0.7–0.8 is considered acceptable, and 0.8 to 0.9 is considered excellent (Mandrekar, 2010; Hosmer and Lemeshow, 2000). The results of this study showed that M2BPGi had low sensitivity and acceptable diagnostic value in diagnosing significant fibrosis, while it had excellent diagnostic value in diagnosing extensive fibrosis. By eliminating each study one by one, the sensitivity analysis showed that the study results were stable.

The results of the quality assessment indicated that many studies were at risk of bias. Due to the disproportionately high percentage of retrospective studies included and the fact that some studies did not specify the interval between blood sampling and liver biopsy, there may be selective bias in patient selection and case flow. In some included studies, the blinding methods were not mentioned, and in addition, the pathological diagnosis results were only completed by one expert, which could lead to risks of bias in the detection of indexes and gold standards. Some studies were determined to have an unclear risk of bias for flow and timing because the derivation of serum samples and performance of liver biopsy were not described. Some studies were considered to be at unclear risk of bias in terms of flow and timing because they did not specify when serum samples were obtained and liver biopsies were performed.

The sources of heterogeneity were explored among the included studies. The levels of M2BPGi vary among different disease types, which may lead to heterogeneity in meta-analysis. According to reports, the levels of M2BPGi in patients with CHC are higher than those in patients with CHB (Nishikawa et al., 2016d). Compared with patients with CHC or CHB, patients with non-alcoholic steatohepatitis (NASH) have the lowest levels of M2BPGi (Shirabe et al., 2018). The difference in gender ratio seems to be one of the sources of heterogeneity, which needs further confirmation. In most studies, each study established an optimal threshold without using a pre-specified threshold. Therefore, there were differences in the optimal cut-off value for staging LF. In addition, this index took different cut-off values in diagnosing LF of different etiologies, which may also contribute to the high degree of heterogeneity in our review. The diagnostic performance of M2BPGi may be affected by geographical bias (7 of 12 studies included were from Japan), which may be due to the first introduction of M2BPGi as a biomarker for LF and its approval for clinical practice happened in Japan (Lian et al., 2022). In addition, we found that age was also an influential factor in the diagnostic performance of M2BPGi.

This study has the following advantages: (1) Compared to previous meta-analyses that usually included studies using liver stiffness measurement (LSM) for staging LF, this study explicitly included studies using liver biopsy as the gold standard for staging LF. (2) This study was not limited to patients with one single cause of LF. (3) The heterogeneity sources of the included studies were analyzed by subgroup analysis and meta-regression analysis. (4) The sensitivity analysis was carried out to prove the stability of the research results. Limitations of this study: (1) There were differences in the design and conduct of the included studies, which may lead to confounding bias. (2) Although this study included patients with various causes of LF, it did not include all causes of LF. (3) The different scoring criteria for staging LF in the included studies were translated into a unified staging method, which was acceptable but resulted in bias. (4) Clinical applicability was limited by the fact that all included patients were from Asian countries, and it is hoped that further studies will include patients from more countries.

## Conclusion

Serum M2BPGi is an effective, non-invasive, and convenient marker for staging significant fibrosis and extensive fibrosis in patients with CHB, CHC, or NAFLD. Despite its limitations, it can be a promising method of dynamic monitoring of the progression or regression of LF.

## Data Availability

The raw data supporting the conclusions of this article will be made available by the authors, without undue reservation.
